# Coordinated force generation of skeletal myosins in myofilaments through motor coupling

**DOI:** 10.1038/ncomms16036

**Published:** 2017-07-06

**Authors:** Motoshi Kaya, Yoshiaki Tani, Takumi Washio, Toshiaki Hisada, Hideo Higuchi

**Affiliations:** 1 Deparment of Physics, Graduate School of Science, University of Tokyo, 7-3-1 Hongo Bunkyo-ku, Tokyo 113-0033, Japan; 2 Department of Human and Engineered Environmental Studies, Graduate School of Frontier Science, University of Tokyo, Kashiwanoha Kashiwa, Chiba 277-0882, Japan

## Abstract

In contrast to processive molecular motors, skeletal myosins form a large motor ensemble for contraction of muscles against high loads. Despite numerous information on the molecular properties of skeletal myosin, its ensemble effects on collective force generation have not been rigorously clarified. Here we show 4 nm stepwise actin displacements generated by synthetic myofilaments beyond a load of 30 pN, implying that steps cannot be driven exclusively by single myosins, but potentially by coordinated force generations among multiple myosins. The simulation model shows that stepwise actin displacements are primarily caused by coordinated force generation among myosin molecules. Moreover, the probability of coordinated force generation can be enhanced against high loads by utilizing three factors: strain-dependent kinetics between force-generating states; multiple power stroke steps; and high ATP concentrations. Compared with other molecular motors, our findings reveal how the properties of skeletal myosin are tuned to perform cooperative force generation for efficient muscle contraction.

## Abstract (web summary)

Skeletal muscle myosin forms large ensembles to generate force against high loads. Using optical tweezers and simulation Kaya *et al*. provide experimental evidence for cooperative force generation, and describe how the molecular properties of skeletal myosins are tuned for coordinated power strokes.

The molecular motors myosin, kinesin and dynein act cooperatively in many cellular processes (that is, muscle contraction, vesicle transport, spindle formation and so on) and drive dynamic cellular activities^1^,^2^,^3^. The mechanical properties of these motors have been extensively investigated at the single-molecule level^4^,^5^,^6^,^7^,^8^,^9^,^10^,^11^,^12^,^13^, revealing the diverse functions of individual motors. Aside from the single-molecule properties, the collective behaviours of these motor proteins have also been investigated *in vitro*^14^,^15^,^16^, in DNA-based molecular scaffolds^17^,^18^,^19^ and in cells^20^,^21^,^22^ to reveal how individual motor functions are integrated into collective motor systems. In general, ensembles of processive motors, which take multiple steps along a filament before dissociating (for example, kinesin 1 and myosin V), exhibit increased run length and processivity as the number of motors increases^18^,^23^. However, the force and velocity of multiple kinesins are not strongly influenced by motor number^15^,^18^. Moreover, the run length and stall force of the non-processive kinesin 5 and 14 are much more sensitive to motor number^18^,^24^ than processive motors. The velocity and force of another non-processive motor, skeletal myosin, also directly correlate with the number of molecules^4^,^5^,^25^. The collective behaviours of motor proteins must be interpreted by considering both single-molecule properties and the mechanochemical and/or chemical interplay among motor proteins^26^,^27^. Thus, an experimental system that enables the detection of the dynamics of individual motors and their ensemble effects must be developed.

Myosin II families, that is, skeletal, cardiac, smooth and non-muscle myosins, form bipolar filaments that interacts with actin filaments, thereby generating actin sliding and force. This arrangement is advantageous for producing high forces and high speeds of shortening since the uniform orientation of the motors avoids the drag effect caused by misaligned motors^28^,^29^. In addition, the drag of negatively strained motors is greatly reduced by the nonlinear elasticity of the myosin head^30^. Thus, these structural properties are partly responsible for the additive force generation of skeletal myosins as the number of motors increases^4^,^5^,^30^,^31^. Mechanical and X-ray diffraction studies of single muscle fibre preparations have provided potential evidence of the coordinated movements of myosin heads during force generation^32^,^33^,^34^. These findings suggest that skeletal myosins interact with actin filaments, not as independent force generators^35^ but as cooperative force generators^32^. However, the mechanism of cooperative force generation has not been resolved at the single-molecule level as detecting the rapid dynamics of individual myosin molecules, particularly at physiological ATP concentrations, is challenging.

Here we used optical tweezer-based microscopy to measure forces generated by synthetic myofilaments with ∼17 interacting molecules, which is the smallest number of molecules required to produce a processively sliding actin filament at 1 mM ATP (∼10% duty ratio for unloaded condition)^31^. Our measurement system permits the detection of actin displacements that are typically 3–7 nm and can occur in <1 ms. If these steps are generated by single myosins, the work performed by the single myosin exceeds the free energy liberated by a single ATP molecule, thus implying that several myosin molecules must act cooperatively during such step generation. Our simulation model predicts that stepwise actin displacements under high loads are generated mainly by the coordination of power strokes among two to three myosin molecules. Combined with the simulation results, our experimental results provide persuasive evidence of coordinated power strokes at the single-molecule level. Our simulation further reveals that the molecular properties of skeletal myosins are specifically tuned to perform cooperative force generation for efficient muscle contractions.

## Results

### Stepwise actin displacements generated by multiple myosins

**Figure 1:**
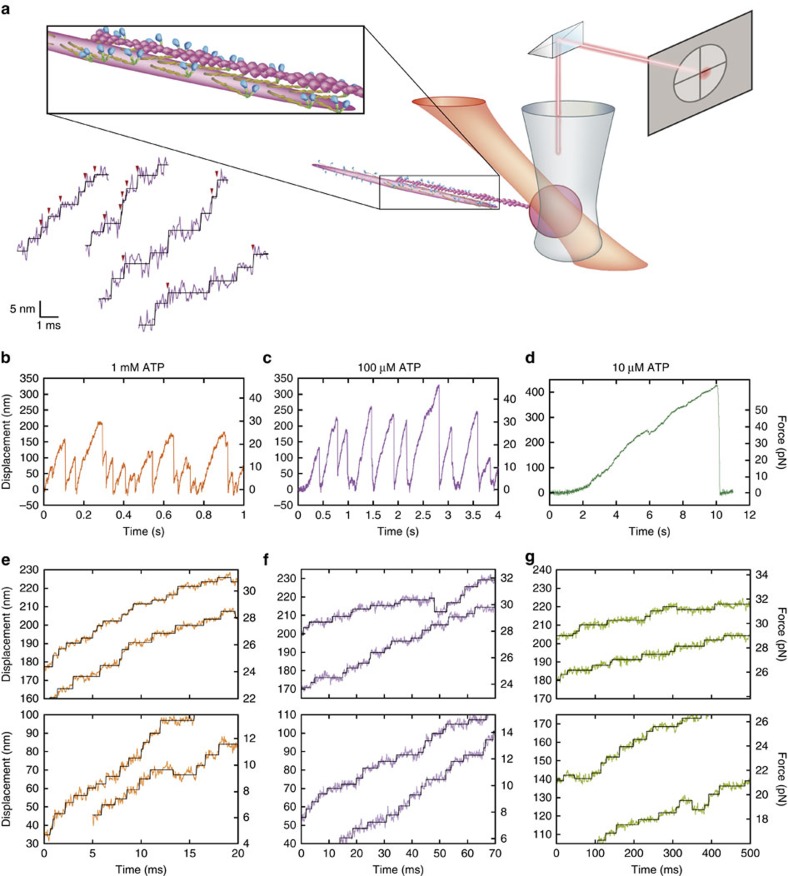
Force generation of myosin-rod cofilaments measured by optical tweezers. (**a**) Schematic of the optical tweezer assay on a synthetic myosin-rod cofilament interacting with an actin filament. A bead coated with streptavidin is attached to a biotinylated actin filament and held above a myosin-rod cofilament. Scattered images of beads diagonally illuminated by a red laser are projected onto a quadrant photodiode sensor. Inset: measured bead displacements with detected steps (black) reveal stepwise displacements that are frequently generated within 1 ms (red arrowheads). (**b**–**d**) Time courses of bead displacement at 1 mM, 100 μM and 10 μM ATP in **b**–**d**, respectively. (**e**–**g**) Stepwise displacements of actin filaments in response to low loads (bottom panels) and high loads (top panels) at 1 mM, 100 μM and 10 μM ATP in **e**–**g**, respectively. The black solid lines represent steps detected by the step-finding algorithm^40^.

**Figure 2:**
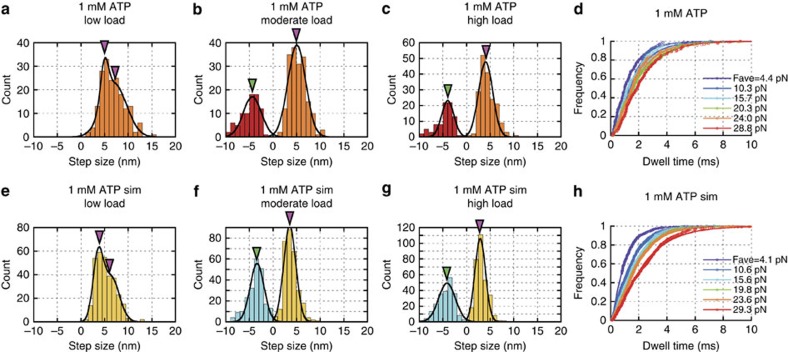
Step size and dwell time of myosin-rod cofilaments. (**a**–**c**) Histograms of step sizes experimentally observed at 1 mM ATP for low loads (mean of 4.4 pN) in **a**, moderate loads (mean of 15.4 pN) in **b** and high loads (mean of 29.3 pN) in **c**. Also see Supplementary Fig. 2 for other ATP concentrations. (**e**–**g**) Histograms of step sizes obtained from the simulation model at 1 mM ATP for low loads (mean of 4.1 pN) in **e**, moderate loads (mean of 15.3 pN) in **f** and high loads (mean of 30.1 pN) in **g**. Also see Supplementary Fig. 2 for other ATP concentrations. (**d**,**h**) Cumulative probability distributions of the dwell time fitted to a double exponential function for experimental data in **d** and simulation data in **h**. The time constants calculated from double exponential fits were used to calculate mean dwell times as shown in Fig. 3g–i. Arrowheads indicate the peak points of single or double Gaussian functions. Step sizes at the peak were used as the mean step size in Fig. 3a–c.

**Figure 3:**
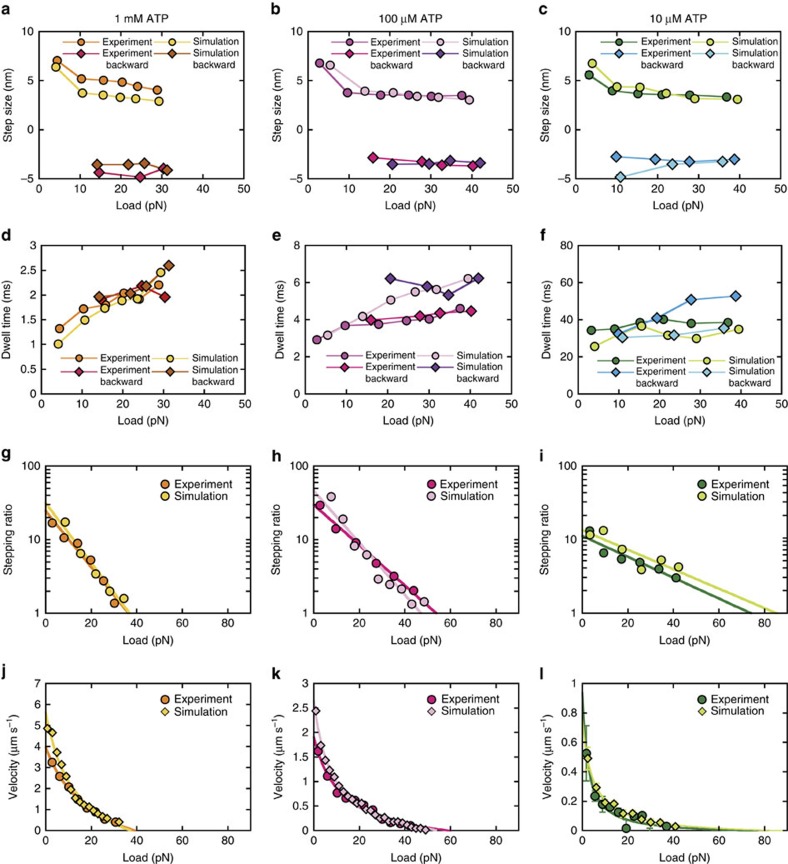
Step properties as a function of load at different ATP concentrations. (**a**–**c**) Mean forward and backward step sizes as a function of load obtained from experiments and the simulation model at 1 mM (experiment: *n*=181 and 84 for forward and backward steps, respectively, from 3 beads; simulation: *n*=649 and 220 for forward and backward steps, respectively, from 12 trials) in **a**, 100 μM in (*n*=863 and 205 for forward and backward steps, respectively, from 3 beads; simulation: *n*=668 and 239 for forward and backward steps, respectively, from 8 trials) in **b** and 10 μM ATP (*n*=335 and 89 for forward and backward steps, respectively, from 4 beads; simulation: *n*=368 and 114 for forward and backward steps, respectively, from 24 trials) in **c**. (**d**–**f**) Mean dwell times obtained from experiments and the simulation model at 1 mM, 100 μM and 10 μM ATP in **d**–**f**, respectively. The abscissas of the fitting functions provided estimates of the stall forces: 36, 54 and 74 pN at 1 mM, 100 μM and 10 μM ATP, respectively, in experiment; 37, 47 and 85 pN at 1 mM, 100 μM and 10 μM ATP, respectively, in simulation. (**g**–**i**) Stepping ratio calculated from experiments and the simulation model at 1 mM, 100 μM and 10 μM ATP in **g**-**i**, respectively. The natural logarithms of the stepping ratio were plotted against the load and fitted to linear functions; the abscissa represents the stall force. (**j**–**l**) Force–velocity (FV) relationships obtained from experiments and the simulation model at 1 mM (experiment: 7 beads; simulation 12 trials), 100 μM (experiment: 10 beads; simulation 8 trials) and 10 μM ATP (experiment: 6 beads; simulation 24 trials) in **j**–**l**, respectively. Each data set was fitted with Hill’s FV equation, (*F*+*a*)(*V*+*b*)=(*F*_0_+*a*)*b*, where *F*_0_, *a* and *b* are stall force and fitting parameters, respectively. The abscissas of the fitting functions provided estimates of the stall forces: 39, 59 and 73 pN at 1 mM, 100 μM and 10 μM ATP, respectively, in experiment; 37, 56 and 86 pN, respectively, in simulation. Error bars represent s.e.m., but most of them are hidden behind symbols.

A 1.2 μm-long myosin-rod cofilament was selected for force measurements such that ∼17 myosin molecules were expected to interact with a single actin filament (Methods), in accordance with our estimate of the number of interacting molecules^30^. Single streptavidin-coated beads (diameter: 400 nm) were attached to biotinylated actin filaments and manipulated by optical tweezers to assess actomyosin interactions (Fig. 1a). The bead displacements showed that forces occasionally increased beyond 30 pN at any ATP concentration (Fig. 1b–d). Because several myosin molecules engaged with actin filaments, fluctuations in actin-bead displacements were substantially suppressed (mean noise level of 2.2 nm against loads of 20–30 pN; Supplementary Fig. 1). In addition, our optical system substantially enhanced the spatiotemporal resolution of the optical tweezers (Supplementary Fig. 1), revealing stepwise displacements with sub-millisecond resolution for 1 mM ATP (Fig. 1a). Specifically, the mean step size decreased from 7 to 4 nm with increasing loads (Figs 2a–c and 3a–c and Supplementary Fig. 2), whereas the dwell times slightly increased in response to loads at any ATP concentration (Figs 2d and 3d–f). Remarkably, two distinctive step sizes were observed for low loads at any ATP concentration (Fig. 2a and Supplementary Fig. 2a,b). In contrast to the forward steps, the backward step sizes were nearly constant for each load (Fig. 3a–c and Supplementary Fig. 3). The stepping ratio, which is the ratio of the numbers of forward and backward steps, under unloaded conditions was higher for 1 mM and 100 μM ATP (forward:backward=30:1) than for 10 μM (forward:backward=10:1; Fig. 3g–i), indicating that myosins generated forward steps more progressively at higher ATP concentrations. The abscissas of the fitting functions provided estimates of the stall forces at each ATP concentration (36, 54 and 74 pN at 1 mM, 100 μM and 10 μM ATP, respectively). The force–velocity relationships observed for myofilaments showed a hyperbolic shape at all ATP concentrations (Fig. 3j–l), as observed for single muscle fibre preparations^36^. If a step size of 4 nm observed in response to a load of 30 pN was to be generated exclusively by one of the interacting molecules (for example, Fig. 1e), the work would be 120 pN nm, which is slightly higher than the free energy of ATP hydrolysis estimated for our experimental conditions (28.5 *k*_B_*T*=117 pN nm; Methods); thus, the mechanical efficiency would be 103%. Although this estimate could slightly change in different buffer conditions, it possibly violates the first law of thermodynamics, thus implying that more than one myosin must contribute to step generations at high loads beyond 30 pN.

**Figure 4:**
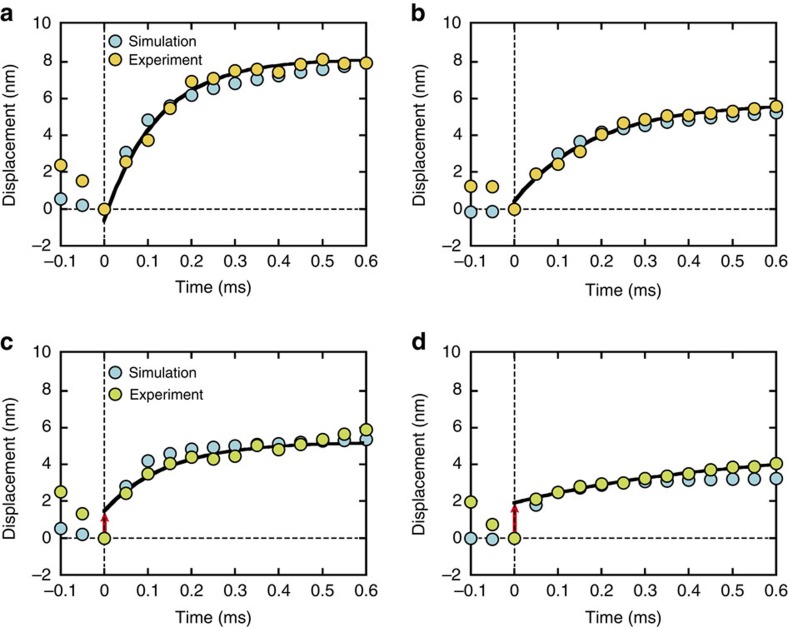
Characteristics of step curves during the rising phase of actin displacement. (**a**,**b**) Ensemble-averaged displacement of actin filaments at 1 mM ATP for low loads (mean of 2.7 pN, *n*=42 for experiment; mean of 2.5 pN, *n*=2,455 for simulation) in **a** and high loads (mean of 23.1 pN, *n*=179 for experiment; mean of 18 pN, *n*=487 for simulation) in **b**. (**c**,**d**) Ensemble-averaged displacement of actin filaments at 10 μM ATP for low loads (mean of 4.3 pN, *n*=51 for experiment; mean of 2.7 pN, *n*=404 for simulation) in **c** and high loads (mean of 28.6 pN, *n*=169 for experiment; mean of 22.7 pN, *n*=545 for simulation) in **d**. The black lines are single exponential curves (*d*=*d*_s_{1−exp[−*t*/*τ*]}+*d*_0_) fitted to experimental data starting from one sampling point after the onset point (*t*=50 μs). The fitting curves extended to *t*=0 pass close to the origin for 1 mM ATP (*d*_0_=−0.6 or 0.4 nm, *d*_s_=8.7 or 5.3 nm and *τ*=120 or 180 μs for low and high loads, respectively), suggesting that the entire rising phase of actin displacement can be approximated by a single exponential event, owing to the nearly coordinated executions of power strokes among multiple myosin molecules (Supplementary Fig. 6a,b). As indicated by the red arrows for 10 μM ATP, the fitting curves deviate from the origin (*d*_0_=1.5 or 1.9 nm, *d*_s_=3.7 or 3.0 nm and *τ*=150 or 495 μs for low and high forces, respectively), indicating that the rising phase can be divided into at least two phases: the first power stroke execution, followed by the second power stroke (Supplementary Fig. 6c,d).

To characterize the steps in the displacement curves, individual steps were aligned at their onset and ensemble-averaged for the first 600 μs (Fig. 4a–d). The rising phase of the steps were fitted well with a single exponential function (Fig. 4). When the best-fit exponentials were extrapolated towards *t*=0, they approached the origin for 1 mM ATP, suggesting that the entire rising phase can be characterized by a single exponential event. Moreover, the best-fit exponentials deviated from the origin for 10 μM ATP, suggesting that the rising phase consist of at least two events.

### Modelling of skeletal myosin filaments

**Figure 5:**
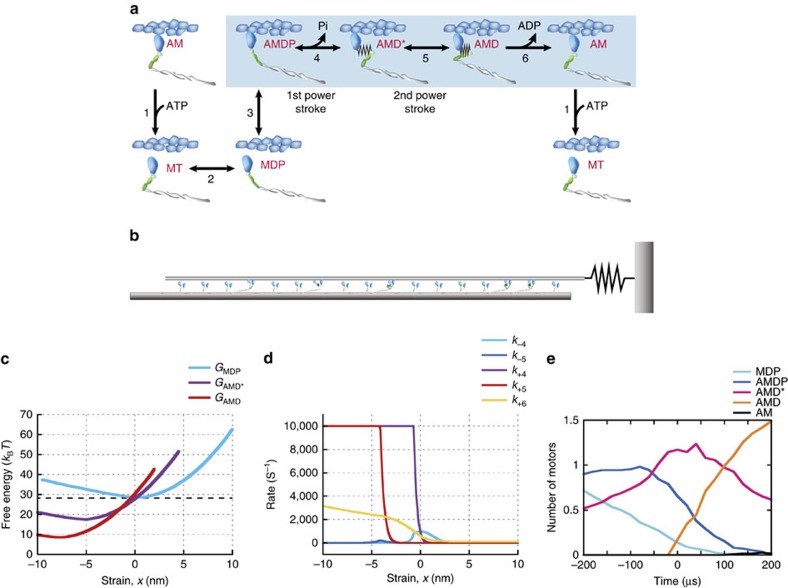
Simulation model for actin–myosin interaction. (**a**) Kinetic scheme for myosin and actin interaction cycles starting from ATP binding to myosin (AM). A, M, T, D and P are actin, myosin, ATP, ADP and Pi, respectively. Two steps of power stroke execution are accompanied at the transitions towards AMD* and AMD states. See details in the Methods section. (**b**) Schematic of 17 myosin molecules interacting with an actin filament connected to the spring, whose stiffness is equivalent to the trap stiffness of 0.16 pN nm^−1^ used in the experiments. (**c**) Free energy profiles of detached (MDP) and attached states (AMD* and AMD) calculated from the nonlinear elasticity of the myosin head as a function of the strain in the myosin head, *x*. The black dashed line represents the total free energy of ATP hydrolysis, Δ*E*_ATP_ (Methods). The difference in free energy minima between MDP and AMD* or AMD* and AMD are given as 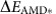 or Δ*E*_AMD_, respectively (Methods and Table 1). (**d**) Strain-dependent rate constants between four force-generating states (*k*_4_, *k*_5_ and k_6_) given by the Boltzmann factor. Also see details in the Methods section. (**e**) The mean population of each state for power stroke myosin within ±200 μs of the onset of the step at 1 mM ATP. Before the onset of the step (*t*<0), myosins primarily populate either the AMDP or AMD* state and then undergo a transition to the AMD* or AMD state associated with the first or second power stroke during step generation (*t*>0).

**Figure 6:**
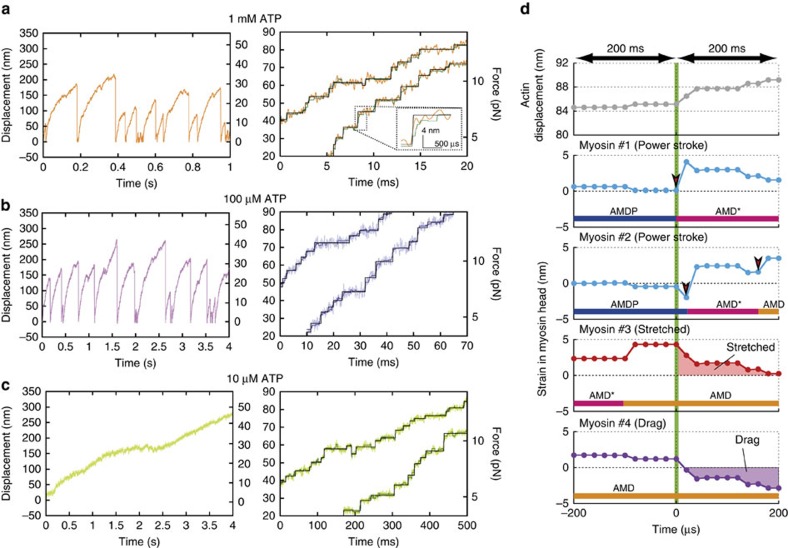
Simulation results for actin–myosin interaction. (**a**–**c**) Time-course data for the displacement of a single actin filament (left column) at 1 mM, 100 μM and 10 μM ATP in **a**–**c**, respectively. Stepwise displacements of actin filaments computed by simulation for low loads (right column). Random noise was added to the original computed steps (different coloured lines) to detect steps (black solid line) by using the step-finding algorithm^40^. The inset in a shows a zoomed-in plot of the box region, which shows a single step (black) generated by contiguous power strokes (green). (**d**) Time course of a single actin step (top) and corresponding strains of myosins bound to actin filaments (bottom) within ±200 μs of the onset of the step, as indicated by the green vertical line. In this example, four myosins, #1–#4, engage with an actin filament during step generation. Myosins #1 and #2 execute multiple power strokes, as highlighted by the red arrowheads, whereas myosins #3 and #4 have already completed power strokes. Thus, they remain bound to actin as a ‘stretched’ or ‘drag’ motor, respectively. Red or purple shaded regions indicate whether an average strain at *t*>0 is positive (stretched) or negative (drag). The horizontal colour bars in each panel indicate transition states.

To gain further insight into the mechanism by which synthetic myofilaments generate stepwise force, we developed a Monte Carlo-based simulation model^37^ consisting of 17 myosin molecules arranged in parallel and interacting with a single actin filament attached to the spring, whose stiffness was equivalent to the trap stiffness of 0.16 pN nm^−1^ used in our experiment (Fig. 5a,b). The transitions between four force-generating states (AMDP, AMD*, AMD and AM) and two detachment states (MT and MDP) were coupled to ATP hydrolysis (Fig. 5a, Table 1 and Methods). Two key features were implemented in our model: (i) strain-dependent transitions between force-generating states; and (ii) two power stroke steps accompanying AMDP and AMD* states. We found that the experimentally observed step size histograms fit well with two Gaussian functions at low loads (with peaks at 4.9 and 7.1 nm for 1 mM in Fig. 2e; 4.2 and 6.8 nm at 100 μM, and 3.8 and 7.2 nm at 10 μM in Supplementary Fig. 2). We assumed that the average position of myosin is −1.2 nm away from the zero strain position before attachment because of the nonlinear elasticity of the cross-bridge^30^. Hence, the first power stroke size of 5.5 nm (refs 38, 39) represented our values well (3.8–4.9 nm+1.2 nm=5.0–6.1 nm), and the second power stroke size was set to 2.5 nm, so that the total power stroke size was 8 nm (ref. 30), which is also consistent with our experimental values (6.8–7.2 nm+1.2 nm=8.0–8.4 nm). The actin filament displacement calculated theoretically closely resembled our experimental data in terms of peak force values and velocities (Fig. 6a–c). The theoretical results also showed stepwise displacements, that is, the steps fluctuated with the addition of random noise before step detection using the step-finding algorithm^40^. The step sizes and dwell times obtained from our simulation model were also consistent with those observed experimentally at any ATP concentration (Fig. 3a–c,d–f), suggesting that our model reasonably reproduces experimental phenomena. Interestingly, the detected steps deviated slightly from the computed steps (Fig. 6a–c), implying that smaller or faster steps were probably hidden within noise, and consequently, only large steps were detected (Supplementary Fig. 4).

### Numbers of coordinated-power stroke myosins

We investigated how myosin molecules generate individual steps by focusing on the 200 μs periods before and after the onset of steps in our model (Fig. 6d). This approach was selected because the rising phase of ensemble displacement data fit a single exponential function with time constants of <200 μs well, suggesting that steps are mostly completed within 200 μs (Fig. 4). In the example shown in Fig. 6d, four myosin molecules attach to an actin filament at different states. Myosin #1 is the motor that initiates the sliding of the actin filament by executing the first power stroke. The sliding of the actin filament then releases the strain of other bound myosin heads because these heads move with the actin filament. Subsequently, myosin #2 is under negative strain and then executes the first power stroke because of a marked increase in the forward transition rate (*k*_+4_) from AMDP to AMD* at *x*<0 (Fig. 5d and Methods). As demonstrated by a series of mechanochemical reactions, we found that consecutive power strokes among multiple myosins are frequently executed within 200 μs during a rising step phase, suggesting that power strokes may be nearly synchronized during step generation. Thus, we regarded the motors that execute rapid sequential power strokes as ‘coordinated-power stroke’ motors. Before the onset of the step, myosins #3 and 4 have already completed the second power stroke (Fig. 6d), and thus, their strain values depend on actin sliding. Myosin #3 remains positively strained throughout a period of 200 μs, and thus this myosin passively contributes to active force generation. Consequently, if myosins are positively strained on average over 200 μs without executing power strokes, they are considered to be ‘stretched’. Conversely, myosins that are, on average, negatively strained over 200 μs (that is, myosin #4; Fig. 6d) are considered ‘drag’ motors. Therefore, we can categorize engaged myosins during the initial 200 μs into three types: coordinated-power stroke; stretched; and drag myosins.

**Figure 7:**
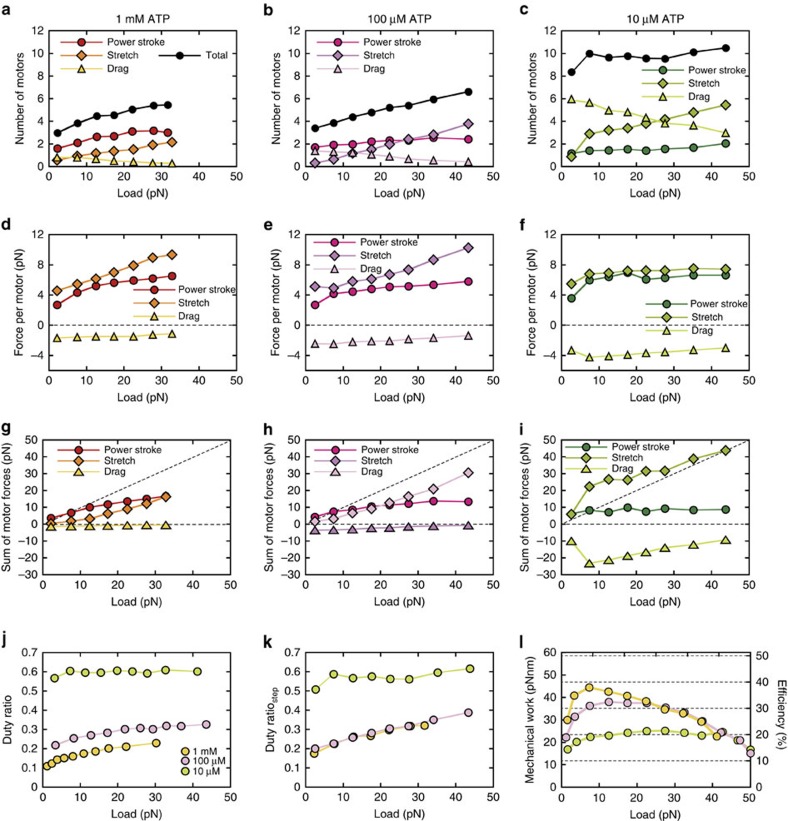
Load-dependent modulation of coordinated-power stroke stretched and drag motors. (**a**–**c**) The mean numbers of coordinated-power stroke, stretched and drag myosin motors as a function of loads obtained from the simulation model at 1 mM, 100 μM and 10 μM ATP in **a**–**c**, respectively. (**d**–**f**) Mean force per coordinated-power stroke, stretched and drag myosin motors as a function of the loads at 1 mM, 100 μM and 10 μM ATP in **d**–**f**, respectively. (**g**–**i**) Sum of forces generated by coordinated-power stroke, stretched and drag myosin motors at 1 mM, 100 μM and 10 μM ATP in **g**–**i**, respectively. Diagonal dashed lines represent the force level, which is equivalent to an applied load. The sum of these force curves perfectly aligns to this line. (**j**,**k**) Duty ratio at 1 mM, 100 μM and 10 μM ATP as a function of load calculated by two methods: a ratio of attachment time to cyclic time in **j** and a ratio of total number of power stroke, stretched and drag motors to 17 motors counted during 200 μs from the onset of detectable steps in **k**. (**l**) Mechanical work and efficiency at 1 mM, 100 μM and 10 μM ATP as a function of load. All mean values are plotted with error bars representing s.e.m., but most error bars are hidden behind symbols.

The number of coordinated-power stroke, stretched and drag motors were counted for 200 μs after the onset of steps and plotted as a function of the load at each ATP concentration (Fig. 7a–c). The number of stretched motors directly correlated with the load, and this relationship was more prominent at lower ATP concentrations (Fig. 7a–c), while the number of drag motors is remarkably high at 10 μM ATP. The number of coordinated-power stroke motors strongly correlated with the load at high ATP concentrations but not at 10 μM ATP. In fact, 53% of the computed steps were generated by coordinated-power stroke myosins at 1 mM ATP, whereas this number fell to 30% of the steps at 10 μM ATP. Thus, these results suggest that the probability of synchronized power strokes increases at higher ATP concentrations, whereas steps are generated primarily by single-power stroke motors at 10 μM ATP.

Interestingly, the load-dependent modulation of coordinated-power stroke motors was virtually abolished when the second power stroke state was eliminated from the original model and replacing it by a single 8 nm stroke (Supplementary Fig. 5). As a consequence, the single-power stroke model could only generate a maximum force of 20 pN (Supplementary Fig. 5), whereas the original two-power stroke model generated peak forces in excess of 30 pN (Fig. 6a), a finding that is consistent with our experimental observations (Fig. 1e). Thus, these results demonstrate the importance of multiple power stroke states to enhance the force output by synchronizing power strokes.

### Power stroke motors at high ATP concentrations

Our simulation model shows that the force per power stroke myosin motor gradually increases as the load increases, and then levels off at 4–6 pN in response to loads exceeding 10 pN (Fig. 7d–f). In contrast, the force per stretched motor, which remains positively strained in the post-power stroke state, proportionally increases with increasing load. The force per drag motor remains nearly constant with changing loads, but is slightly higher at lower ATP concentrations. The contribution of the sum of forces generated by coordinated-power stroke, stretched and drag motors to the total force output depends on the ATP concentration (Fig. 7g–i). Forces generated by coordinated-power stroke motors primarily contribute to the total force throughout the entire range of loads at 1 mM ATP, whereas forces generated by stretched motors primarily contribute to the total force in response to increasing loads at 100 and 10 μM ATP. Particularly for low-mid loads at 10 μM ATP, forces generated by stretched motors appear to be higher than the corresponding external loads (that is, forces exceed the diagonal dashed line; Fig. 7i) because of high drag forces. These differences between force contributions of the different motors were attributed to the changing coordination of these motors at 1 mM and lower ATP concentrations (Fig. 7a–c). The work per myosin motor moderately increased around the mid-load range at any ATP concentration and increased with higher ATP concentrations (Fig. 7l).

### Step curves at low and high ATP concentrations

The number of coordinated-power stroke motors involved in step generation markedly differed between 1 mM and 10 μM ATP (Fig. 7a–c), implying that the characteristics of simulating step displacements might differ at 1 mM and 10 μM ATP, as observed experimentally (Fig. 4). The ensemble-averaged step displacement curves were consistent with the corresponding experimental curves (Fig. 4). The differences obtained between 1 mM and 10 μM ATP concentrations can be explained by our simulation results, which indicated that two to three myosin molecules execute a power stroke nearly simultaneously at 1 mM ATP (Fig. 7a and Supplementary Fig. 6a,b), in what appears to be a single exponential event. However, a single myosin is primarily responsible for the rising phase of the step at 10 μM ATP (Fig. 7c and Supplementary Fig. 6c,d), which appears to consist of two events: (i) the rapid execution of the first power stroke, followed by (ii) a load-dependent second power stroke with *k*_+5_ of ∼10,000 s^−1^ (Fig. 5d). Overall, these characteristics of step curves obtained from our simulation model were consistent with those obtained experimentally at 1 mM and 10 μM ATP, thus supporting our working hypothesis that steps are generated by contiguous power strokes executed by multiple myosin molecules at 1 mM, but probably not at 10 μM ATP.

## Discussion

Our experimental set-up enabled the detection of stepwise displacements of actin filaments with mean step sizes of 3–7 nm every 1–2 ms at 1 mM ATP (Fig. 1a). The observed inverse correlation between step size and load (Fig. 2a–c) was consistent with our previous results^30^ except for two distinctive step sizes observed at low loads (Fig. 2a and Supplementary Fig. 2a,b), which can be revealed by high spatiotemporal resolution of our measurement system (Supplementary Fig. 1). The power stroke size of 5.5 and 2.5 nm used for the simulations resulted in similar peak values of the Gaussian functions as those observed experimentally (Fig. 3a–c), suggesting that skeletal myosins may have two distinct power strokes^32^. We tested different combinations of step size in the simulation model (that is, 2.5+5.5 nm and 4+4 nm: the first stroke size of 2.5 or 4 nm followed by the second stroke size of 5.5 or 4 nm; Supplementary Fig. 7) and found that the power stroke size of 5.5+2.5 nm is the best with experimental results among these step sizes. The force per power stroke (4–6 pN) and the duty ratio (10–20%) at 1 mM ATP (Fig. 7d,j) were consistent with the values estimated from single-fibre mechanical measurements^31^,^41^,^42^ for unloaded shortening and isometric contractions of single fibres. Interestingly, at 1 mM ATP, the duty ratio calculated as a ratio of attachment time to cyclic time is slightly lower than that calculated as a ratio of total number of power stroke, stretched and drag motors to 17 motors counted during 200 μs from the onset of detectable steps (yellow circles in Fig. 7j,k). The difference is due to a systematic increase in coordinated-power stroke motor particularly for large detectable steps at 1 mM ATP. The force per power stroke was also consistent with the average isometric value of 5.7 pN observed in single molecules^43^. Moreover, the force–velocity curves of the myosin ensemble (Fig. 3j–l) showed a hyperbolic shape with curvature values (that is, *a*/*F*_0_ in Hill’s force–velocity equation) of 0.14–0.53 for 1 mM and 100 μM ATP, which was within the range of reported values for skeletal muscle^36^, suggesting that an ensemble of 17 myosin molecules is sufficient to reconstitute muscle contractile properties at high ATP concentrations^44^. The discrepancy in unloaded velocity between experiment and simulation model could be due to the difference in the number of interacting motors available at the beginning of force generation. In experiments, positions of trapped bead were adjusted relative to myosin-rod cofilaments so that an actin filament was overlapped at least with one-half of myosin-rod cofilament, however a less number of motor can bind to an actin filament because of actin filament fluctuation, while in simulation, all the myosin molecules are assumed to be available throughout the entire force generation. Our simulation results (not presented) also suggested that a range of decrease in velocity (Fig. 3j–i) could be due to a loss of two to three interacting molecules. The variability in peak force observed in a series of bead displacements in experiments and simulations (for example, Figs 1b,c and 6a,b) could be due to a stochastic nature of actomyosin interactions, which force generation depends on how many of available motors are recruited for interaction. In summary, our experimental system, combined with the simulation model, allows investigation of not only the molecular properties of skeletal myosin but also the corresponding ensemble effects, which shed light on the molecular mechanisms of muscle contraction.

The experimental and simulation data showed that the stepping ratio of a myofilament is 10:1 for unloaded conditions at 10 μM ATP (Fig. 3i), which is similar to that of single myosins at 1 mM ATP in our simulation model. Interestingly, this ratio was 30:1 at 1 mM and 100 μM ATP (Fig. 3d,e). Although 30:1 is lower than the 200:1 ratio obtained for the highly processive kinesin 1 motor^46^,^47^, our results revealed that the ensemble effect of skeletal myosins enhances unidirectional movements at high ATP concentrations.

The steps detected in response to loads exceeding 30 pN could not have been generated exclusively by a single myosin molecule because the mechanical work exceeds the free energy of ATP hydrolysis. In fact, the work per myosin never exceeds the free energy of ATP hydrolysis (Fig. 7l), and the efficiency given as a percentage of work relative to the free energy of ATP hydrolysis was ranged between 15 and 38% (Fig. 7l), suggesting that the results were consistent with those estimated from single-fibre studies^45^. The work per myosin tends to be lower for lower ATP concentrations (Fig. 7l), since higher numbers of drag motors are involved in force generations (Fig. 7c), resulting a decrease in mechanical work due to high negative strains (that is, *x*_3_<<0; Methods and equation (8)). The work per myosin was also calculated for our previous experimental condition with four interacting molecules at 20 μM ATP (ref. 30) and was similar to these results (not presented), suggesting that individual myosins function energetically in a consistent way, however an ensemble effect remarkably changes mechanical properties (for example, force generation, dwell time, stepping ratio and so on) of myosin ensembles with different interacting molecules. In addition, a twofold increase in the dwell time in response to loads of ∼30 pN (Fig. 3d–f) is unexpected if myosins generate forces independently each other. Thus, these results imply that mechanical actions of myosins are coupled possibly by sharing changes in strain of myosin head via a sliding of actin filament (for example, Fig. 6d). Duke^48^,^49^ predicted the stepwise displacements of actin filaments by myosin motor ensembles because of power stroke coordination among multiple motors due to coupling of mechanochemical reactions. Combined with these results, the stepwise displacements observed in the current study can be attributed to the coordination of power strokes among multiple myosin molecules.

To gain molecular insight into the mechanism of coordinated power strokes among myosin motors, a simulation model was implemented with two key features: (i) a strain-dependent kinetics between force-generating states; and (ii) a two-step power stroke that accompanies the AMDP and AMD* states. The model predicts coordinated power strokes among myosin molecules at high loads and at high ATP concentrations (Fig. 7a–c). To increase the possibility of coordinated power stroke, several myosins must be briefly ‘stuck’ in either the AMDP state accompanied by the first power stroke or the ADM* state accompanied by the second power stroke (Fig. 6d). As the load increases, coordination is further reinforced by decreasing the transition rates in the positive strain direction (*k*_+4_ and *k*_+5_ in Fig. 5d). The sliding of the actin filament caused by a power stroke myosin then releases the strains of the ‘stuck’ myosin heads, triggering a rapid transition to the next state that is accompanied by a power stroke (for example, Fig. 6d). When the ATP-binding rate (*k*_+1_) is lower than the rates of *k*_+4_ and *k*_+5_ (for example, *k*_+1_=40 s^−1^ at 10 μM ATP), *k*_+1_ becomes the rate-limiting step. Thus, bound myosins are mainly populated in the post-power stroke AM state, and the possibility of a coordinated power stroke is substantially decreased, while these bound myosins more likely become drag (Fig. 7c). The mean number of coordinated-power stroke motors is 1–1.5 for the loads up to 30 pN at 10 μM ATP (Fig. 7c), suggesting that individual steps are mainly generated by either one- or two-power stroke motors (Supplementary Fig. 6d,e). To overcome such less-coordinated force generations with drag effects, ‘stretched’ myosins make a larger contribution to active force generation (Fig. 7c,i). Another distinctive feature of force generation at 10 μM ATP is a significant increase in force due to detachments of drag motors. Drag motors tend to be more negatively strained at lower ATP concentrations (Fig. 7f), resulting a significant increase in force and step when detached (Supplementary Fig. 6d). These results demonstrate that the ATP-binding rate must be reasonably high to increase the possibility of power stroke coordination (for example, *k*_+1_>5,000 s^−1^ for [ATP]>1 mM). Consequently, the strain-dependent transitions associated with the first or second power stroke are the rate-limiting steps under high loads (an order of ∼10 to ∼100 s^−1^ at *x*>0; Fig. 5d). This strain-dependent kinetics might be an effective strategy to enhance the force output in skeletal myosin ensembles, particularly at high ATP concentrations, when myosins cannot remain bound to actin filaments for a long period of time but can increase the force output by increasing the number of coordinated-power stroke motors.

Our working hypothesis, which states that power stroke coordination is more effectively modulated at high compared to low ATP concentrations, appears to be consistent with our experimental data (Fig. 4). As predicted by our computational model, the ensemble-averaged curves of the experimentally observed steps showed a single exponential increase in steps at 1 mM ATP because several myosins execute a power stroke within hundreds of microseconds of each other, resulting in a single event with time constants of 120–180 μs (Fig. 4a,b and Supplementary Fig. 6a,b). Moreover, the actin filament displacement curves obtained at 10 μM ATP primarily consisted of an initial rapid event followed by a slower single event, which may represent two power stroke steps (Fig. 4c,d and Supplementary Fig. 6c,d). Hence, the difference in the step displacements between the 1 mM and 10 μM ATP conditions may be attributed to differences in the number of power stroke motors involved in step generation.

Multiple power stroke steps may increase the possibility of power stroke coordination. Stall force in the single-power stroke model (∼20 pN) was smaller than that obtained for the two-power stroke model (>30 pN), because the former has a decreased probability of power stroke coordination due to the loss of the second power stroke (Supplementary Fig. 5a). We found that the single-power stroke model can generate a similar amount of force as that observed in the two-power stroke model by increasing the load-dependent properties (that is, increasing the characteristic distance value, *δ*, or decreasing the forward transition rates, *k*_+4_ and *k*_+5_, at *x*>0, experimental procedures). However, the stepwise movements were found to be too slow, compared to those observed in our experiment. Moreover, the efficiency of the single-power stroke model was lower than that of the two-power stroke model throughout the entire range of loads, as reported by others^38^, because the single-power stroke model requires a greater number of stretched motors due to the lower chance of power stroke coordination (Supplementary Fig. 5a), resulting in higher ATP consumption. These results suggest the importance of multiple power stroke steps for efficient force generation.

The theoretical framework of ensemble behaviours among multiple kinesins combined with experimental results^50^ suggested that the motors, whose velocities drop rapidly with increasing load (for example, skeletal myosin in Fig. 3j–l), appear to have more capability of additive force generation in their ensembles than the processive motor kinesin, since they can develop load-sharing configurations more readily. This may be attributed to two unique features of skeletal myosin: (i) its non-linear elasticity, which minimizes the drag effects of post-power stroke motors; and (ii) its coordinated power stroke, which results in the cooperative generation of force among myosins. Here we show that this mechanical coupling among myosin motors may be enhanced by three factors: the strain-dependent kinetics between force-generating states; multiple power stroke steps; and high ATP concentrations. Hence, these molecular properties of skeletal myosins are specifically tuned to perform coordinated force generation for efficient muscle contractions.

It is trivial to test whether coordinated force generations is essential for *in situ* muscle fibre contractions because the compliance of sarcomere structures in series with myofilaments (for example, ∼50 pN nm^−1^ (ref. 51)) is more than two orders of magnitude smaller than the trap stiffness of 0.16 pN nm^−1^ in our experiments so that coordinated force generations could be a minor effect under the sub-nanometre strain release in sarcomere. Thus, the simulation was run to investigate whether coordinated force generation is still effective in a low series compliance of sarcomere by implementing the trap stiffness of 50 pN nm^−1^ and 75 myosin-interacting molecules (Supplementary Fig. 8). The simulation results showed that a single actin filament is displaced maximally up to 6–7 nm, corresponding to 300–350 pN, which is a typical force range of isometric contractions observed in single muscle fibre^31^,^51^. As expected, step sizes are much smaller due to the low series compliance. However, coordinated force generation occurs occasionally when a series of rapid steps (for example, top 10% of large actin displacements=0.4–1.5 nm within 200 μs) is generated during force development, in which the elastic deformation of actin/Z-disc complex occurs. For example, in Supplementary Fig. 8b, the simulation results showed that six motors consecutively execute a power stroke triggered by a sub-nanometre sliding of actin filament, which is initially driven by another motor, while another 13 motors generate forces as either stretched or drag myosins (not presented). Since the kinetic rate constant (*k*_4+_) of first power stroke markedly increases at small negative strain values (for example, *k*_4+_>2,000 s^−1^ at *x*≤−0.2 nm in Fig. 5d), power stroke coordination is conceptually effective during force development in *in situ* isometric contractions. Meanwhile, a smaller fraction of actin sliding mostly appears in plateau force, in which a few number of power stroke motors are coordinated (Supplementary Fig. 8c).

The concept of coordinated power strokes was originally proposed to explain the quick force recovery observed in response to step releases up to 15 nm imposed on contracting single muscle fibres^31^,^32^,^38^,^52^. In contrast to isometric contractions, these step releases increase the chance of power stroke coordination, even in a low series compliance of sarcomere. Power strokes are synchronized by step releases to accomplish quick force recovery responses within 1–2 ms, while the slow force recovery phase is accounted for by the repeated cycles of myosin motor attachment and detachment. In accordance with some of those models^31^,^32^,^38^,^52^, in our model myosins are allowed to perform multiple power stroke steps without detaching from actin. In this scenario, the transition rates between different states are dependent on strain (for example, *k*_+4_ and *k*_+5_). However, the functional implications of quick release experiments for regular muscle activity are tentative at best because the rapid length/tension releases performed within 1–2 ms are much faster than the timeframe of the physiological regime of muscle contraction. In the current study, we confirmed the molecular properties proposed in the 1971 cross-bridge model at the single-molecule level. On the basis of our findings, we further argue from a molecular point of view that they may be uniquely essential for skeletal myosin to increase the possibility of power stroke coordination, thus allowing ensembles of myosin molecules to cooperatively generate force in response to high loads.

## Methods

### Simulation model

To identify the crucial factors that may affect cooperative force generation among myosin molecules, our simulation model was designed to feature six actomyosin interaction states. The rate constants for reactions between AM and AMDP states were determined on kinetics studies of actomyosin in solution^53^,^54^ (Fig. 5a). The equilibrium constant of ATP binding, *K*_1_, was taken as 400 μM (*k*_−1_=2,000 s^−1^, *k*_+1_=5 × 10^6^ M^−1^ s^−1^) and the rate constants of ATP hydrolysis, *k*_−2_ and *k*_+2_, were assumed to be 10 and 100 s^−1^, respectively. For the transition between MDP and AMDP, the attachment rate of myosin with actin filament, *k*_+3_, is govern by the Boltzmann equation as follows:


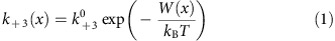



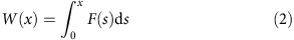


where 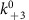 is the constant of 300 s^−1^ and *W*(*x*) is the elastic potential energy of myosin head with the nonlinear stiffness (*k*_m_=2.8 pN nm^−1^)^30^, depending on the strain, *x*, as follows:


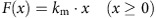



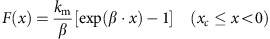



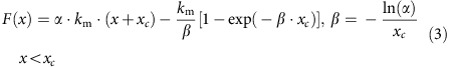


where *x*_*c*_ and *α* are the constants of 4.35 nm and 0.05, respectively. This rate function is skewed towards the negative strain side because of lower stiffness of myosin head at *x*<0. The detachment rate, *k*_−3_, is set to be 30 s^−1^. These constant values were determined as the most appropriate values for our study since they depend on ionic strength and temperature^53^,^55^. The equilibrium constants *K*_4_ and *K*_5_ are given by the Boltzmann equation:


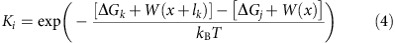


where *j* and *k* are AMDP and AMD*, or AMD* and AMD, respectively. *l*_*k*_ is the power stroke length (5.5 or 2.5 nm for *k*=AMD* or AMD), which ensures that the total power stroke length is 8 nm (ref. 30). 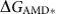
and Δ*G*_AMD_ are the free energy of 10.3 *k*_B_*T* from MT to AMD* and 8.4 *k*_B_*T* from AMD* to AMD, respectively. These values were determined by comparing the force outputs with the corresponding experimentally observed values (Fig. 1b–d) and appear to be within the reasonable ranges of those assigned in other model^56^ (Methods and Table 1). *W*(*x*) is the potential energy with nonlinear elasticity as a function of myosin strain, *x* (ref. 30). The following rate functions were assigned to satisfy equation (4):


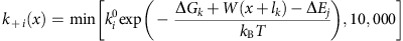



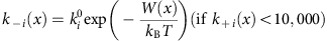



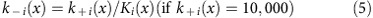


where *k*_*i*_^0^ is the constant of 1,400 or 560 s^−1^ at *i*=4 or 5, respectively. All constants are similar to the ranges of those assigned in other models^38^,^56^ (Methods and Table 1). Note that *k*_*i*_^0^ and Δ*G*_*i*_ are typically combined as one parameter (that is, 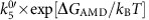)^38^ and thus, this value is also listed in Methods and Table 1.

ADP release may depend on load^38^,^57^,^58^ and was given by the following relation:


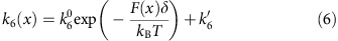


where *F* is the motor force, and 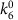 and 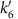 are the rate constants (500 and 100 s^−1^, respectively). *δ* is a characteristic distance of 1.6 nm that determines the load dependency. These constants were originally obtained from the literature^38^,^58^ and were slightly modified to fit our experimental data. All parameter values were summarized in Methods and Table 1.

The total free energy of ATP hydrolysis is given by


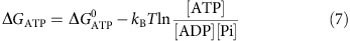


where 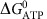 is the standard free energy of ATP hydrolysis of −13.2 *k*_B_*T* (refs 54, 59) and [ATP], [ADP] and [Pi] are the molar concentrations of ATP, ADP and Pi. In our experimental condition, the ATP solution maximally contains 1.5% of ADP and Pi contamination and therefore, it is assumed here that [ADP] and [Pi] are 15 μM, respectively, for 1 mM ATP. The ATP consumption rate at the myosin concentration of 28 pM in our experimental condition is ∼10 nM h^−1^. Thus, the reduction of ATP concentration in a 30–40 min experimental session is negligibly small compared to the experimental concentration of 1 mM, 100 μM and 10 μM ATP. The additional ATP concentration from actin filaments is expected to be only 50 nM and negligibly low. Therefore, 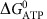 is simply calculated to be 28.5 *k*_B_*T* for 1 mM ATP by equation (7) (Fig. 5c). In our simulation model, the consumption of energy in the force-generating states (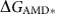
+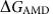) is 18.7 *k*_B_*T* so that 66% of the total free energy of ATP hydrolysis is used during force generations (Fig. 5c). This value is similar to the consumption ratio of 71% in other model^56^.

Persuasive evidence has indicated that myosin and actin filaments are non-Hookean^51^,^60^. However, thick and thin filaments were assumed to be rigid in our model because the nonlinear compliance of these filaments may not be essential for our experimental conditions, in which synthetic myofilaments are fixed to a glass surface. The stiffness of myofilament immobilized on substrate (7 pN nm^−1^) is three to four times higher than that of the myosin head^30^. The stiffness of the 2–3 μm actin filament length used in our experiment (20–50 pN nm^−1^) is several orders of magnitude higher than the myosin stiffness^51^,^61^. These filaments’ compliances are negligibly small in comparison with the trap stiffness of 0.16 pN nm^−1^ used in the current study. Thus, these filaments were assumed to be rigid in all simulations. We also assumed that myosin could bind anywhere on actin. To simulate a more realistic interaction between myosins and actins, an axial periodicity of myosin (that is, 43 nm) and actin filaments (that is, 36 nm) should be taken into account. However, the X-ray diffraction study suggested that diffraction patterns from a contracting muscle are primarily governed by the axial periodicity of myosin filament^62^. Moreover, the simulation model suggested that the effect of mismatch between a periodicity of myosin and actin filaments is averaged over time during actomyosin interactions^38^. Thus, these characteristics may not significantly affect on force generations. Meanwhile, the modelling of discrete actin-binding sites (5.5 nm pitch) would limit displacements of actin driven by power stroke of myosin^63^. In our simulation, the thermal fluctuation of myosin head is −5 to 4 nm because of the nonlinear elasticity^30^, causing a variation of step size. This range is similar to the target zone of three actin monomers (−5.5 to 5.5 nm), which is an accessible range of single-myosin bindings^63^. Therefore, our simulation results should not be distinctively different from a more realistic model of actin-binding site. The effects of Ca^2+^ regulatory proteins troponin and tropomyosin were not considered in the current model.

Mechanical work per myosin was calculated as a sum of changes in potential energy at two phases: phase (i) from the instant of the first power stroke execution to the instant before the second power stroke execution and phase (ii) from the instant of the second power stroke execution to the instant before detachment. Work per myosin is given by


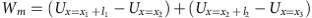



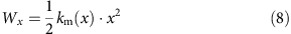


where *x*_1_, *x*_2_ and *x*_3_ are motor strains at the instant before the first power stroke, second power stroke and detachment from actin filaments, respectively. *k*_m_(*x*) is the stiffness of myosin head, which is nonlinearly changed as a function of strain, *x* (Methods and equation (3))^30^. *l*_1_, *l*_2_ and *W*_*x*_ are the first and second power stroke size, 5.5 and 2.5 nm, respectively, and the potential energy of myosin head.

### Protein preparation

Skeletal myosins and G-actin were purified from rabbit fast-twitch muscle^30^. To synthesize myosin-rod cofilaments, myosins and tetramethylrhodamine isothiocyanate-labelled rods were mixed at a molar ratio of 4:1. The mean myosin-rod cofilament length of 1.19±0.19 μm (*n*=10) was selectively picked for force measurements so that the experimental conditions with the giving mixing ratio give the ∼17±3 myosin-interacting molecules, according to our previous study^30^. The mixing solution was rapidly diluted three times to decrease the ionic strength of the standard assay buffer (20 mM PIPES (pH 7.2), 5 mM MgSO_4_ and 2 mM EGTA) from 600 to 100 mM KCl.

### Step size and force measurements

Flow chambers (∼8 μl volume) were prepared using 24 mm × 32 mm and 18 mm × 18 mm coverslips with 30 μm-thick double-sided tape. The chambers were incubated with 0.1 mg ml^−1^ casein, followed by a 3 μg ml^−1^ myosin-rod cofilament solution. The solution was then replaced with a mixture of 400 nm-diameter streptavidin-coated polystyrene beads (Polysciences, Inc.), 2 nM biotinylated phalloidin-tetramethylrhodamine isothiocyanate (PHD-TRITC) labelled actin, an oxygen scavenger system, 1 μM phalloidin, 1 mM creatine phosphokinase, 2 mM creatine phosphate, and 10 μM, 100 μM or 1 mM ATP. Once the bead was trapped by optical tweezers (1,064 nm, 800 mW, Spectra Physics, Inc.) and attached to a 2–3 μm actin filament by manipulating a three-axis custom stage controlled by a water hydraulic micro manipulator (Narishige, Inc.), the actin filament was placed near a myosin-rod cofilament that was fixed on a glass surface. The actin filament was positioned such that it overlapped with at least half of the myosin-rod cofilament. Although we cannot control the actin filament position accurately relative to the myosin-rod cofilaments, the effect of actin placement on the force generation of correctly oriented myosins is negligible in our experimental set-up. All experiments were conducted at 25 °C.

The dark-field images of the beads, which were diagonally illuminated by a focused red laser (685 nm, 50 mW, CrystaLaser), were projected onto quadrant detector photodiodes and recorded at a sampling rate of 20 kHz with a bandwidth of 10 kHz. The displacement of beads was corrected by setting the compliances of the bead–protein complex to ∼5%. The steps were detected on the basis of the bead displacement data with a low-pass filter at a bandwidth of 5 kHz using a step-finding algorithm^40^. The position of trap laser was fixed so that the force driven by the myosins was calculated by multiplying the bead displacement by the trap stiffness (0.16 pN nm^−1^). Sliding velocities for the force–velocity relationships were calculated as a time derivative of bead’s displacement curves low-pass filtered by a zero-phase Butterworth filter at the cutoff frequency of 200 Hz.

### Data availability

Data supporting the findings of this study are available within the article (and its Supplementary Information file), or are available from the authors on reasonable request.

## Additional information

**How to cite this article:** Kaya, M. *et al*. Coordinated force generation of skeletal myosins in myofilaments through motor coupling. *Nat. Commun.* **8**, 16036 doi: 10.1038/ncomms16036 (2017).

**Publisher’s note:** Springer Nature remains neutral with regard to jurisdictional claims in published maps and institutional affiliations.

## Supplementary Material

Supplementary Information

## Figures and Tables

**Table 1:** Parameter values in simulation model.

	**Parameter value**	**Reference values and sources**
*k*_+1_	5 × 10^6^ M^−1^ s^−1^	4–5 × 10^6^ M^−1^ s^−1^ (refs 4, 54)
*k*_−1_	2,000 s^−1^	2,000 s^−1^ (ref. 54)
*k*_+2_	100 s^−1^	100 s^−1^ (refs 54, 56)
*k*_−2_	10 s^−1^	10 s^−1^ (ref. 54)
*k*_+3_	60 s^−1^	60 s^−1^ (ref. 30)
*k*_−3_	300 s^−1^	300 s^−1^ (ref. 30)
*l*_*k*_ (AMD*→AMD)	5.5 nm	Determined by fit
*l*_*k*_ (AMD→AM)	2.5 nm	Determined by fit
Δ*E*_ATP_	28.5 *k*_B_*T*	Estimated by equation (4)
Δ*E*_AMD*_	10.3 *k*_B_*T*	14 *k*_B_*T* (ref. 56)
Δ*E*_AMD_	8.4 *k*_B_*T*	1.7 *k*_B_*T* (ref. 56)
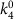	1,400 s^−1^	6,000 s^−1^ (ref. 56)
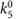	5,768 s^−1^ (a)	4,600–7,800 s^−1^ (ref. 38)
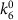	500 s^−1^	350 s^−1^ (ref. 58)
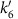	100 s^−1^	Determined by fit
*δ*	1.6 nm	0.7–1.8 nm (refs 38, 58)

^(a)^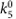 was calculated as 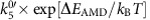 (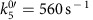 defined in our model) and compared to the corresponding values found in other models since other models^38^,^58^ define 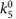, instead of two parameters, Δ*E*_AMD_ and 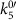.
