# Supplementary material for: Coordinated force generation of skeletal myosins in myofilaments through motor coupling
Source: Nat Commun. 2017 Jul 6;8:16036. doi: 10.1038/ncomms16036 (PMC5504292; doi:10.1038/ncomms16036)
Supplement: Supplementary Information [file ncomms16036-s1.pdf]

Type of file: PDF
Size of file: 0 KB
Title of file for HTML: Supplementary Information
Description: Supplementary Figures and Supplementary Reference

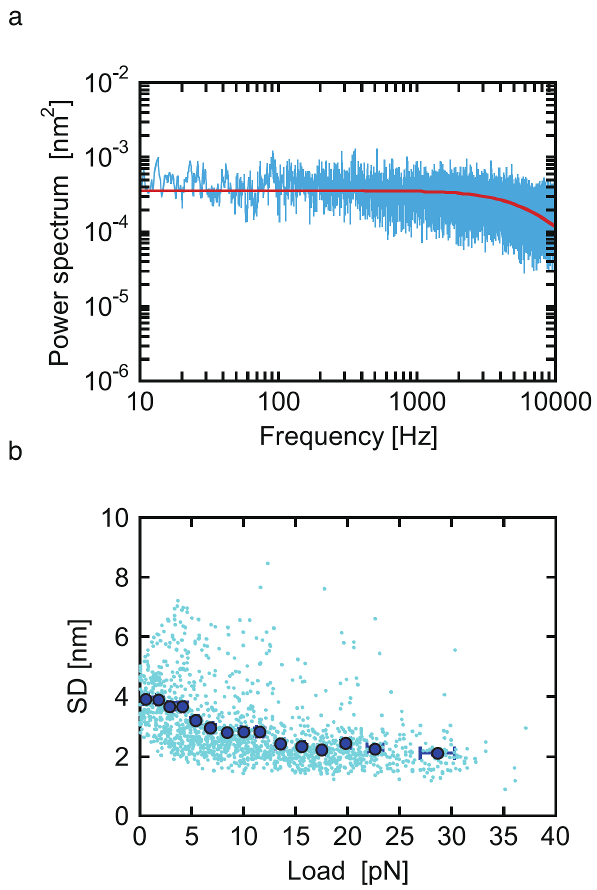

Supplementary Figure 1 | Noise level of bead displacement data measured by optical tweezers. (**a**) Typical power spectrum of the bead position (diameter = 400 nm) trapped by optical tweezers at a sampling rate of 20 kHz. The spectrum is fitted with the power spectrum function, $PSD(f) = \psi_0/(1+(f/f_c)^2)$, where $\psi_0 = (2k_BT\gamma)/k^2$ and γ and k are the viscous drag coefficient and trap stiffness of 0.16 pN nm^{-1}, respectively, and $f_c = k/2\pi\gamma$ is the corner frequency. The fitting function (red) estimates the corner frequency of 6980 Hz, which corresponds to a trap stiffness of 0.15 pN nm^{-1} and a temporal resolution of 23 µs. (**b**) The noise levels (standard deviations, SD) of trapped bead displacement traces during actomyosin interactions from 7 displacement traces at 1 mM ATP were plotted as a function of load. Results clearly show an average decrease in the noise level from 4 to 2 nm as loads increase up to 30 pN. Light blue dots are individual SD values calculated every 10 ms, and dark blue circles are their mean values; error bars represent s.e.m., but most error bars are hidden behind symbols.

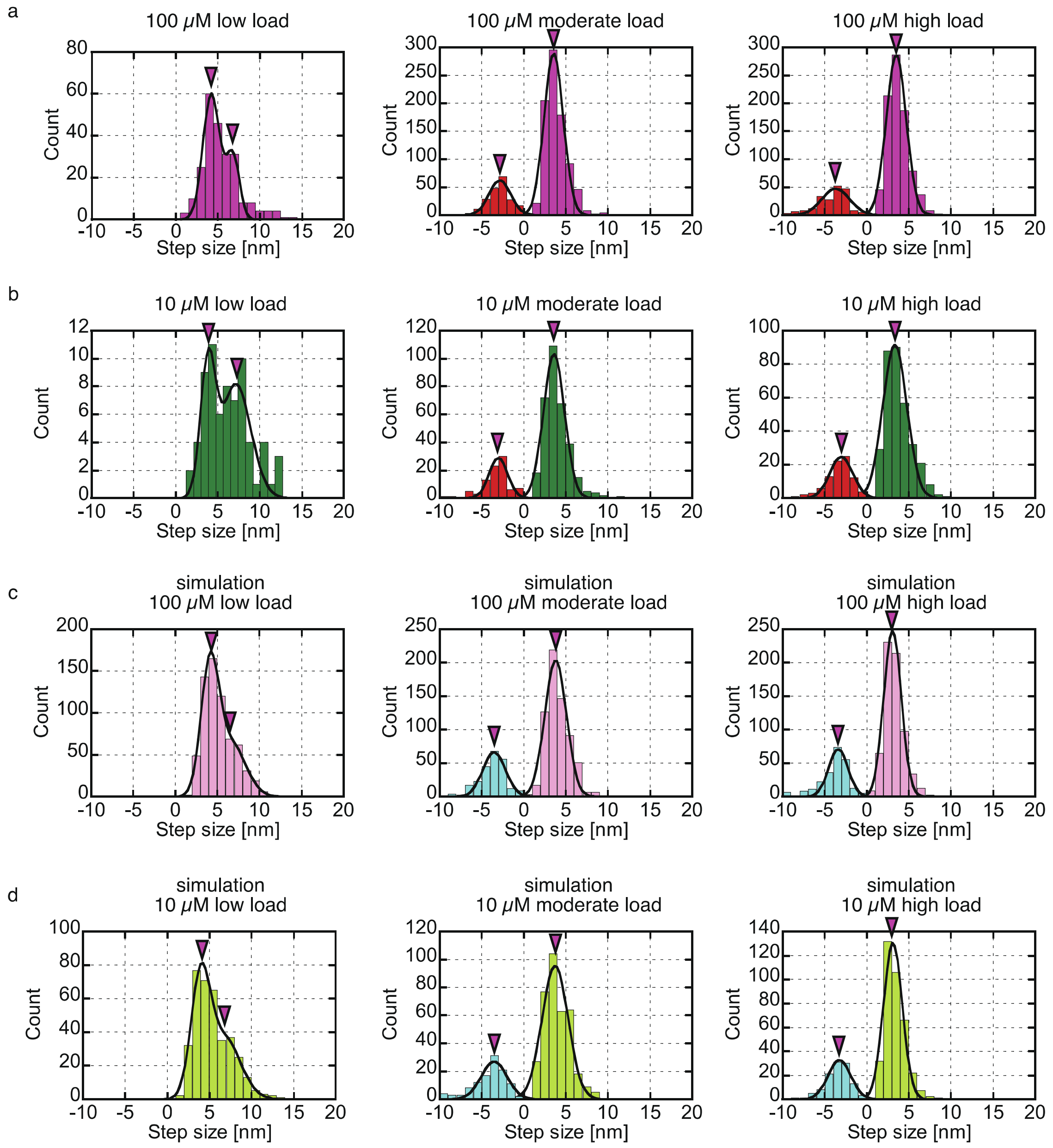

Supplementary Figure 2 | Step size histograms for experimental and simulation data. (**a-d**) Histograms of step size for low (left), moderate (middle) and high loads (right) obtained from experiments at 100 µM in **a** and 10 µM ATP in **b** and simulation at 100 µM in **c** and 10 µM ATP in **d**. For forward step sizes, the data were fitted with the function that provided the best fit, either a single or double Gaussian function. A double Gaussian function with two peaks is typically used for low loads, as shown by the arrowheads. All backward step sizes are fitted with single Gaussian functions. These peak values are used to show mean step sizes in **Fig. 3a-c**.

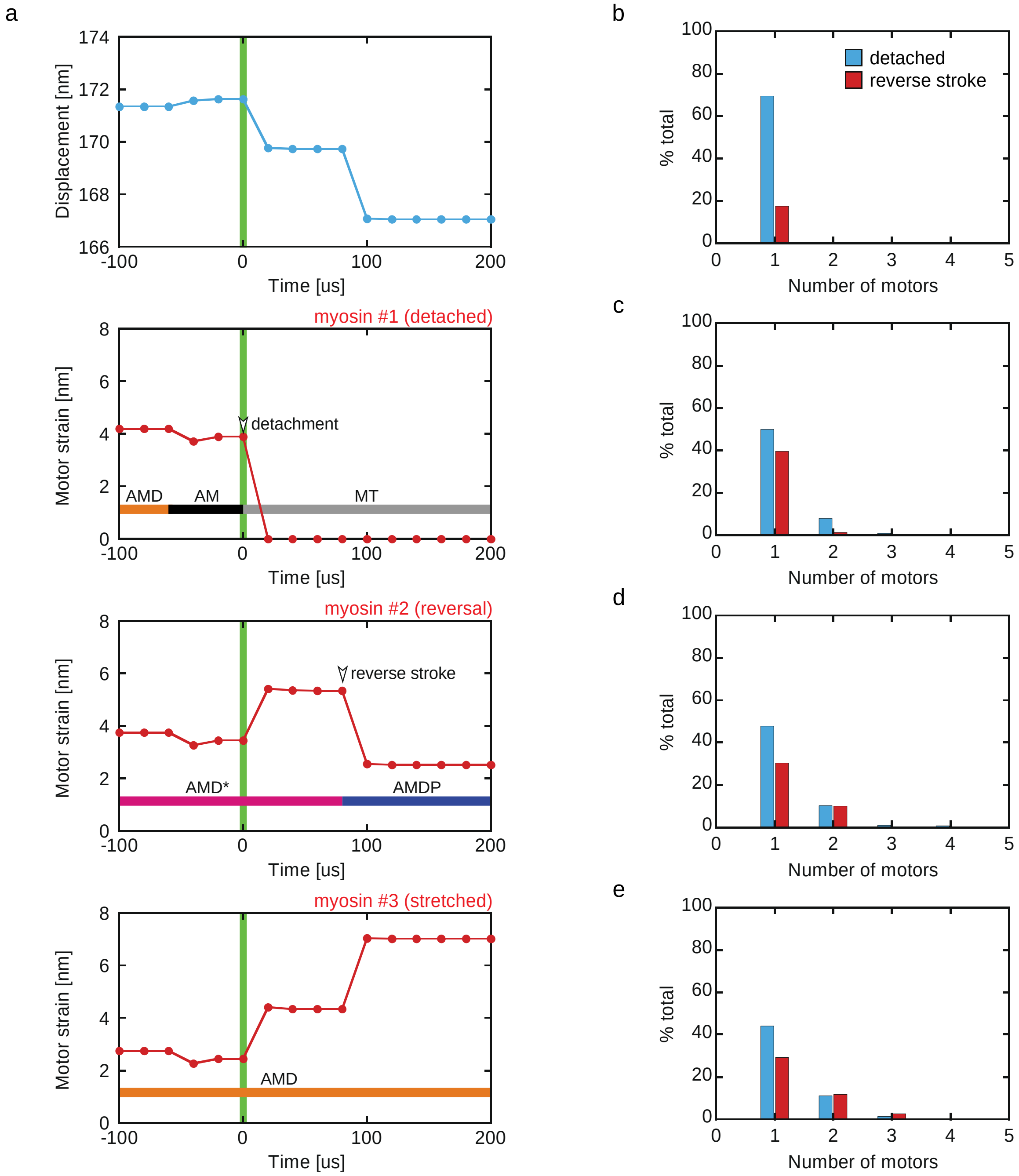

Supplementary Figure 3 | Backward steps are caused by detached and reverse stroke myosin motors. (**a**) Example of mechanochemical reactions during a backward step against a high load of 23 pN at 1mM ATP. Displacement of actin filament shows a backward step (top). This backward step was initially caused by the detachment of myosin #1 (2nd top) at the AM state, followed by the reverse stroke of myosin #2 (3rd top) due to a backward transition from the ADP* to AMDP state, whereas myosin #3 (bottom) remained bound to an actin filament as a stretched

motor. The rate constant of the backward transition from the AMD* to the AMDP state (k_{-4}) increases to exceed that of the forward transition to the AMD state (k_{+4}) when myosin's strain is positive (i.e., x > 0, **Fig. 5d**). Hence, myosin #2 executes reversal of power stroke in response to a large positive strain. Note that the reverse stroke size was set to -5.5 nm, but the strain of myosin #2 does not show an instantaneous decrease of 5.5 nm because the strains of the bound myosin head are determined by the force-balance equation between the bound myosins and load. (**b-e**) Occupation of the number of detached or reversal stroke motors causing backward steps in response to a mean load of 7.2 pN (n = 20) in **b**, 15.7 pN (n = 214) in **c**, 24.9 pN (n = 585) in **d** and 33.0 pN (n = 154) in **e** at 1 mM ATP. In general, the detachment of myosin primarily causes backward steps for low loads, whereas both detachment and reverse strokes of myosin result in almost equal backward steps for high loads, as shown in **a**. The size of the backward step (d_{back}) can be approximately estimated as $d_{back} = F_{detach}/(N_{bound} \times k_m)$, where F_{detach}, N_{bound} and k_m are a loss of force due to myosin detachment, the numbers of bound myosin molecules and the myosin stiffness (k_m = 2.9 pN/nm), respectively. In general, both F_{detach} and N_{bound} are small for low loads and large for high loads, resulting in a nearly constant backward step size, as shown in **Fig. 3a-c**.

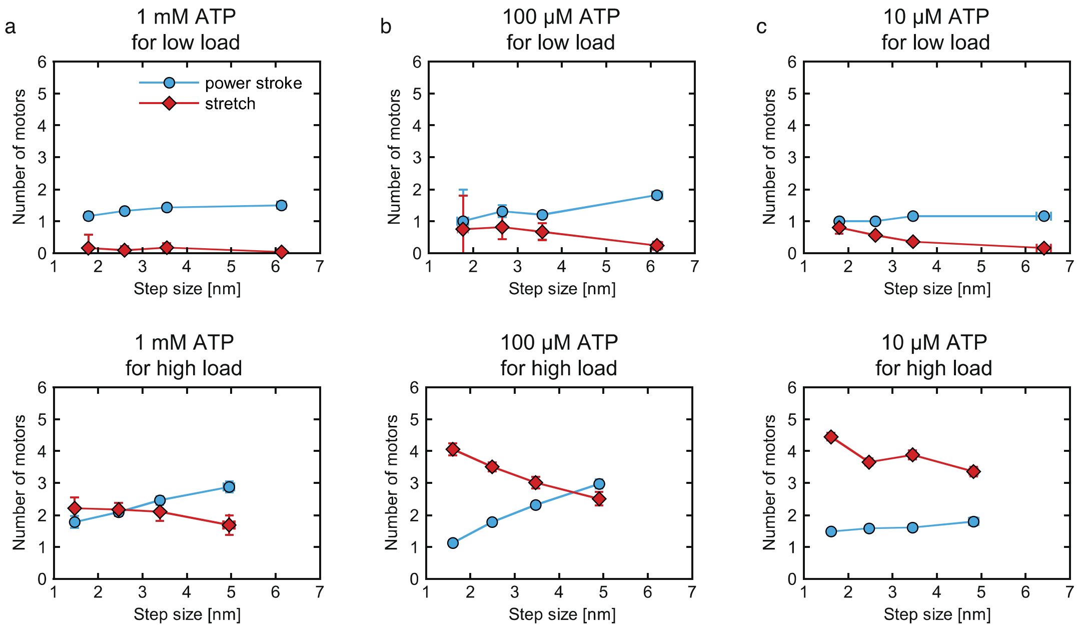

Supplementary Figure 4 | Modulations of the number of synchronous power stroke and stretched motors in response to low and high loads. (**a-c**) The number of synchronous power stroke and stretched motors plotted as a function of the step size for low loads (top) and high loads (bottom) at 1 mM in **a**, 100 μM in **b** and 10 μM ATP in **c**. In general, the number of synchronous power stroke motors (blue) directly correlates with the step size. In contrast, the number of stretched motors (red) inversely correlates with the step size. This trend is more prominent for higher loads. Thus, these results clearly demonstrate that large step sizes can be generated by recruiting more synchronous power stroke motors, and these changes are important to enhance distinctive force generation in response to high loads. Note that stretched motors are defined as a motor whose average strain is positive within 200 μs of the onset of the step (e.g., “myosin #3” in **Fig. 6d**).

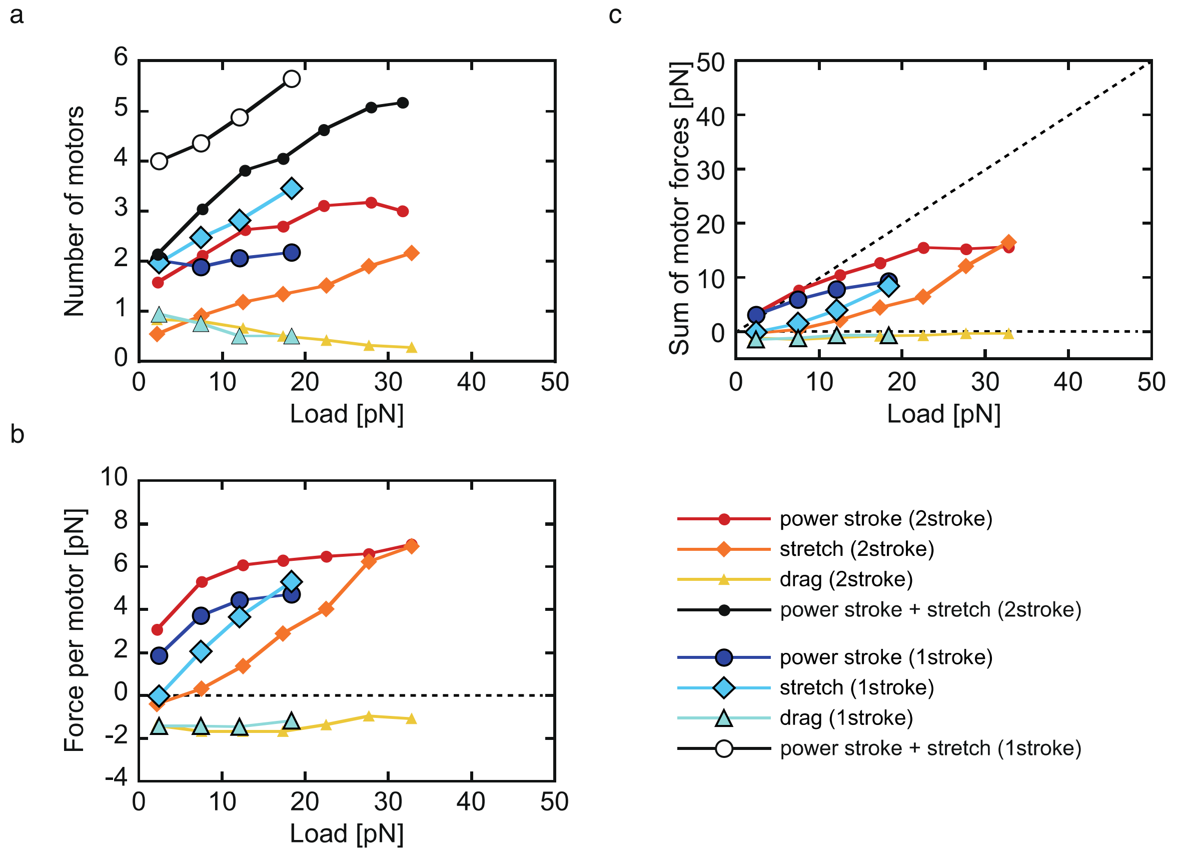

Supplementary Figure 5 | Comparison of the force capacity in the myosin ensemble between one and two power stroke steps. (**a**) Comparison of the number of coordinated power stroke, stretched and drag motors during a step generation period of 200 μs as a function of the load at 1 mM ATP between the original model with two power stroke steps (5.5 + 2.5 nm, two power stroke model) and the single power stroke model (8 nm, one power stroke model). The number of power stroke motors remains nearly constant at approximately 2 molecules for the one power stroke model (blue circles), whereas this number increases from 1.5 to 3 molecules for the two power stroke model (red dots). In contrast, the number of stretched motors is higher for the one power stroke model (blue diamonds) than for the two power stroke model (orange diamonds). The sum of the number of power stroke and stretched motors is higher for the one power stroke model (white circles) than for the two power stroke model (black dots) throughout the entire load range, implying that the one power stroke model requires higher ATP consumptions to generate the same amount of load. (**b**) Force per coordinated power stroke, stretched and drag motors as a function of load. The force per power stroke is lower

for the one power stroke model (blue circles) than that for the two power stroke model (red dots), whereas the force per stretched motor is higher for the one power stroke model (blue diamonds). (**c**) Sum of forces generated by coordinated power stroke, stretched and drag myosin motors as a function of load. Because of the load-independent modulation of recruiting coordinated power stroke motors for the one power stroke model, the force capacity is limited to a maximum of 20 pN for the one power stroke model, whereas it exceeds 30 pN for the two power stroke model. The data for the two power stroke model are the same as those in **Fig. 7a**, **d**, and **g**.

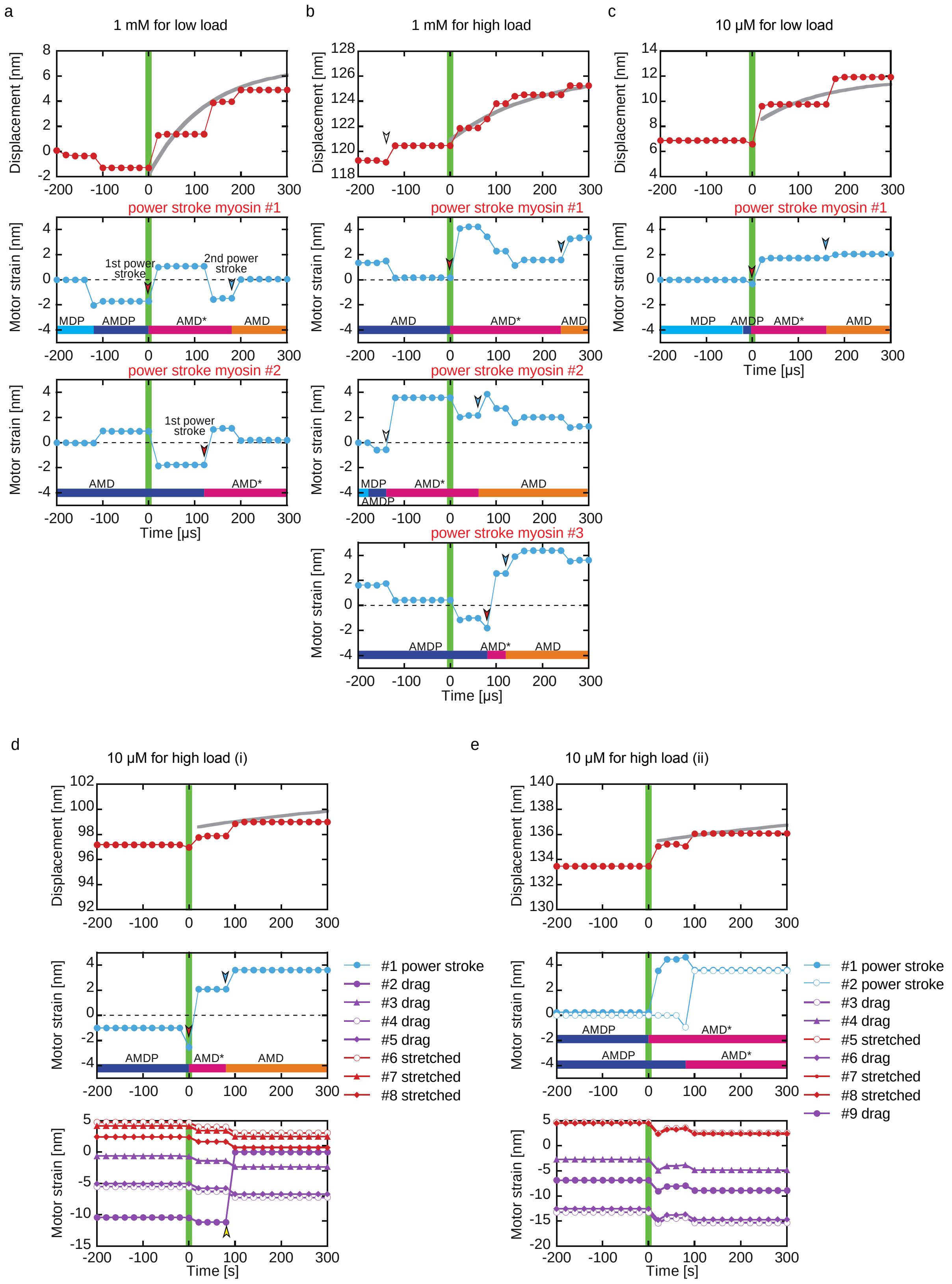
a
1 mM for low load
b
1 mM for high load
c
10 μM for low load
Displacement [nm]
Motor strain [nm]
power stroke myosin #1
power stroke myosin #2
power stroke myosin #3
1st power stroke
2nd power stroke
MDP
AMDP
AMD*
AMD
Time [μs]
d
10 μM for high load (i)
e
10 μM for high load (ii)
#1 power stroke
#2 drag
#3 drag
#4 drag
#5 drag
#6 stretched
#7 stretched
#8 stretched
#2 power stroke
#5 stretched
#6 drag
#9 drag
Time [s]

Supplementary Figure 6 | Example of molecular mechanochemical events during step generation. (**a, b**) At 1 mM ATP, Actin filament displacements (top panels, red) are plotted with single exponential curves (gray) fitted with ensemble-averaged displacement curves in **Fig. 4**. These displacement curves are reasonably similar to these single exponential curves, demonstrating that the rising phase of the displacement curve generated by a synchronous power stroke of 2-3 myosins can be approximated as a single exponential event with time constants of 120-180 μs (see **Fig. 4a, b**). The white arrowhead in top panel (**b**) indicates the instant of the isolated power stroke, demonstrating a subtle increase in the actin displacement due to the non-cooperative power stroke executed by myosin #2. (**c, d**) Actin displacements (top), power stroke motor strains (middle) and stretched and drag motor strains (bottom) at 10 μM ATP. The displacement curves generated primarily by a single power stroke myosin consist of two components: an initial rapid step increase generated by the first power stroke (red arrow heads), followed by a slower single exponential event generated by a second power stroke (blue arrow heads) with time constants of 150-490 μs (see **Fig. 4c, d**). The yellow arrowhead in bottom panel (**d**) indicates the instant of drag motor detachment, resulting an increase in force. In this case, the detachment coincides with the second power stroke execution. The increase in force by detachment and power stroke is 9 pN, which is offset by the decrease in force by the release of strain in stretched and drag motors, resulting a net increase of 0.16 pN in force and thus, 1 nm in step. (**e**) The displacement curve generated by two power stroke myosins at 10 μM ATP. The green vertical lines indicate the onset of the step, and the red and blue arrowheads indicate the onsets of the first and second power strokes, respectively.

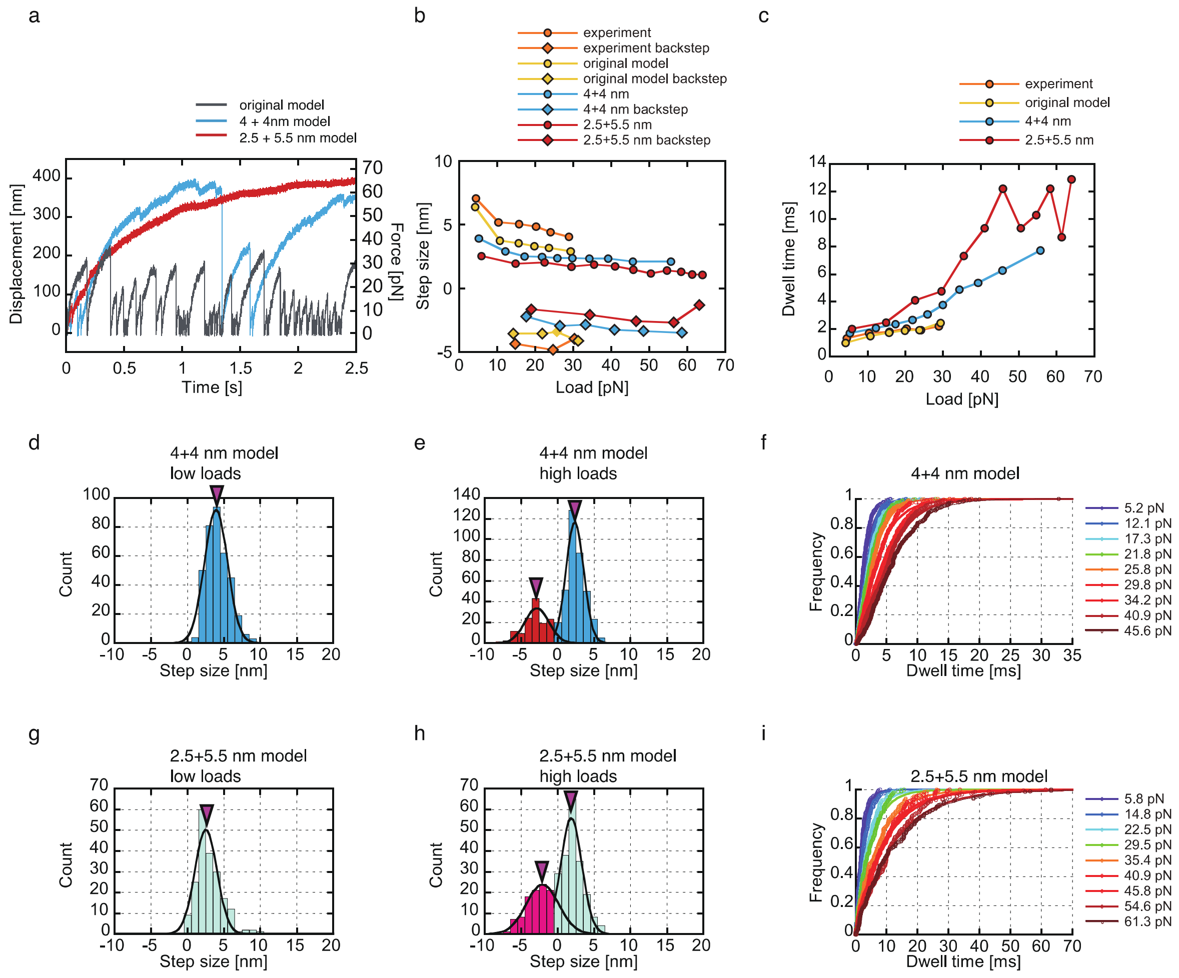

Supplementary Figure 7 | Stepping properties and force generations of myosin ensembles with different sizes of two-step power stroke. (**a**) Time courses of actin displacement generated by the original model (5.5 + 2.5 nm, gray) and the model with two steps of 4 + 4 nm (blue) and the model with two steps of 2.5 + 5.5 nm (red) at 1 mM ATP. The rising phases of displacement curve for the 4+4 nm model are similar to that for the original model, while the 2.5+5.5 nm model shows the distinctively slower curve. Both models generated the forces far exceeding 40 pN, which was never observed in experiments, since the force outputs are enhanced by the decrease in the first power stroke size, which accelerates the transition from AMDP to AMD* state accompanied by the first power stroke. Consequently, the populations of force-generating (AMD*) myosins are increased, resulting higher duty ratio and processivity against high loads, compared to the original model. (**b**) Mean forward and backward step sizes as a function of load obtained from experiments (orange), the original simulation model (yellow) and the model with two steps of 4 + 4 nm (blue)

and 2.5 + 5.5 nm (red) at 1 mM ATP. The forward step sizes obtained from the 4 + 4 nm and 2.5 + 5.5 nm models are smaller than those obtained from experiments and the original model across the entire load ranges. (**c**) Mean dwell times as a function of load obtained from experiments (orange), the original simulation model (yellow) and the model with two steps of 4 + 4 nm (blue) and 2.5 + 5.5 nm (red) at 1 mM ATP. The dwell times obtained from the 4 + 4 nm and 2.5 + 5.5 nm models are longer than those obtained from experiments and the original model. This tendency is more prominent at higher loads (i.e., F > 20 pN). Note that the dwell times for the 2.5+5.5 nm model appear to be fluctuating against the loads beyond 50 pN, since slopes of displacement curve level off and thus, it is hard to detect smaller steps (1-3 nm) against the similar noise levels, leading inaccurate step detections. (**d, g**) Histograms of step sizes obtained from the 4 + 4 nm model (mean of 5.2 pN) in **d** and the 2.5 + 5.5 nm model (mean of 5.8 pN) in **g** at 1 mM ATP for low loads. Unlike experiments and the original model, a single Gaussian function is the best to fit the histograms of step sizes for low loads, since the transition from AMDP to AMD* state accompanied by the first power stroke is accelerated by decreasing the first power stroke size and thus, the step sizes are primarily distributed close to the first power stroke size (e.g., 4 or 2.5 nm for low loads in **b**). (**e, h**) Histograms of step sizes obtained from the 4 + 4 nm model (mean of 34.2 pN) in **e** and the 2.5 + 5.5 nm model (mean of 35.4 pN) in **h** at 1 mM ATP for high loads. The peak points of single Gaussian functions (arrow heads) were used to calculate mean step sizes as shown in **a**. (**f, i**) Cumulative probability distributions of the dwell time fitted to a double exponential function for the 4 + 4 nm in **f** and the 2.5 + 5.5 nm model in **i** at 1 mM ATP. The time constants calculated from double exponential fits were used to calculate mean dwell times as shown in **b**. In summary, these results clearly demonstrate that the stepping properties and force outputs generated by the model with two-step power stroke of 4 + 4 nm or 2.5 + 5.5 nm are distinctively different from experimental results.

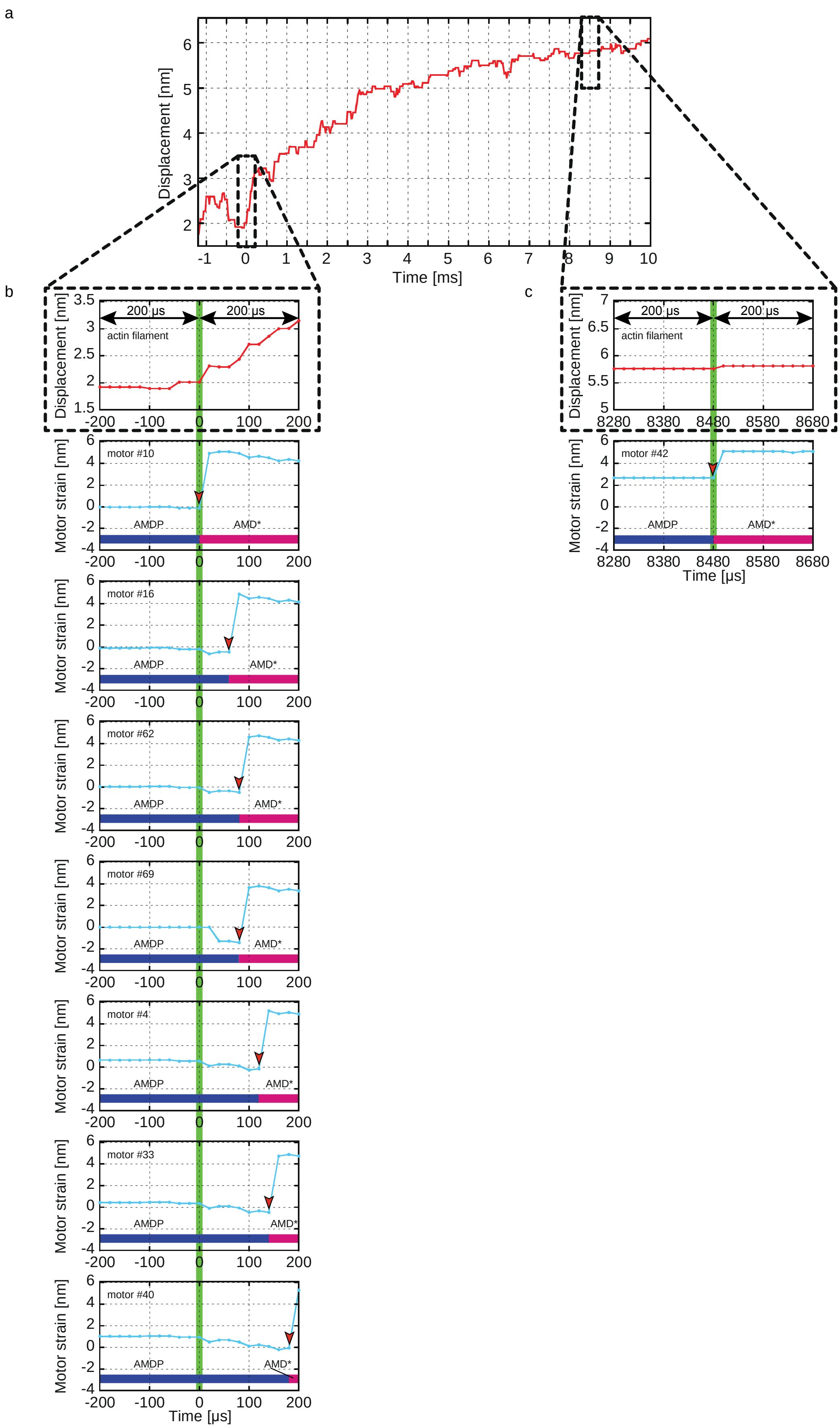
a
Displacement [nm]
Time [ms]
b
c
200 µs
200 µs
actin filament
Displacement [nm]
Motor strain [nm]
motor #10
motor #16
motor #62
motor #69
motor #4
motor #33
motor #40
motor #42
AMDP
AMD*
Time [µs]

Supplementary Figure 8 | Simulation results for *in situ* muscle fiber isometric contraction. (**a**) Time course of actin displacement generated by 75 myosin molecules interacting with a single actin filament, which is connected by the spring (50 pN/nm), corresponding to a stiffness of the actin filament and Z-disc. The left dashed square area in the top panel (**a**) shows a rapid increase in actin displacement (i.e. d > 1 nm witin 200 μs), which occasionally occurs, and is expanded in the top panel (**b**), with the corresponding changes in power stroke motor stains shown in the bottom panels (**b**). Another 13 stretched or drag motors (not presented) also contribute to force generations. The green line represents the onset of a step. Myosin #10 initiates actin sliding, which triggers the execution of power strokes in other myosins (i.e., myosins #16, #62, #69, #4, #33 and #40), as highlighted by red arrowheads. The results demonstrate that sequential power stroke execution can be triggered, even by sub-nanometer actin filament sliding in an *in situ* muscle fiber because of high kinetic rates of k_{+4} at small negative strain values (i.e., -2 < x < 0 nm, **Fig. 5b**). Meanwhile, the right dashed square area in top panel (**a**) shows a smaller fraction of actin sliding (i.e. d ≈ 0.05 nm within 200 μs), which is mostly observed in isometric contractions, and is expanded in the top panel (**c**). In this case, a single power stroke motor (bottom panel **c**) and 26 stretched or drag motors (not presented) contribute to force generations. A total of 75 interacting molecules were chosen to represent the number of myosin molecules interacting with a single actin filament in the sarcomere based on the following assumption: 150 myosin molecules are distributed along one half side of a myofilament, and a single actin filament is surrounded by three myofilaments arranged in a triangular configuration (sum of inner angles = 180 deg), giving the approximation of 75 myosins (= 150 myosin × 180 deg / 360 deg). This estimate is consistent with the estimated value of 84 molecules from the single fiber mechanical measurements[1].

Reference

1. Piazzesi, G. et al. Skeletal muscle performance determined by modulation of number of myosin motors rather than motor force or stroke size. *Cell* **131**, 784-95 (2007).
